# Influence of resin modified glass ionomer cement incorporating protein-repellent and antimicrobial agents on supragingival microbiome around brackets: an *in-vivo* split-mouth 3-month study

**DOI:** 10.7717/peerj.14820

**Published:** 2023-02-06

**Authors:** Yansong Ma, Chengjun Su, Hao Yang, Hockin H.K. Xu, Yuxing Bai, Yan Xu, Xiaoxia Che, Ning Zhang

**Affiliations:** 1 Department of Orthodontics, School of Stomatology, Capital Medical University, Beijing, China; 2 Center for Stem Cell Biology & Regenerative Medicine, University of Maryland School of Medicine, Baltimore, MD, USA; 3 Marlene and Stewart Greenebaum Cancer Center, University of Maryland School of Medicine, Baltimore, MD, USA; 4 Department of Advanced Oral Sciences and Therapeutics, School of Dentistry, University of Maryland, Baltimore, MD, USA

**Keywords:** Orthodontic adhesive, Oral microbiome, Protein repellent, Antimicrobial activity, Enamel demineralization

## Abstract

**Objective:**

To explore the influence of resin modified glass ionomer cement (RMGIC) adhesives containing protein-repellent and quaternary ammonium salt agents on supragingival microbiome, enamel and gingival health around brackets.

**Materials and Methods:**

Ten patients (21.4 ± 3.5 years) about to receive fixed orthodontics were enrolled in this study. Unilateral upper teeth bonded with RMGIC incorporating 2-Methacryloyloxyethyl phosphorylcholine (MPC) and Dimethylaminohexadecyl methacrylate (DMAHDM) were regarded as experimental group (RMD), while contralateral upper teeth bonded with RMGIC were control group (RMGIC), using a split-mouth design. Supragingival plaque was collected from both groups before treatment (T0), and at 1 month (T1) and 3 months (T2) of treatment. High-throughput sequencing was performed targeting v3–v4 of 16S rRNA gene. *Streptococcus mutans* and *Fusobacterium nucleatum* quantification was done by qPCR analysis. Bracket failures, enamel decalcification index (EDI), DIAGNODent scores (Dd), plaque index (PI) and gingival index (GI) were monitored at indicated time points.

**Results:**

Within 3 months, alpha and beta diversity of supragingival plaque had no difference between RMGIC and RMD groups. From T0 to T2, the relative abundance of *Streptococcus* depleted in RMD but remained steady in RMGIC group. *Streptococcus*, *Prevotella*, and *Fusobacterium* became depleted in RMD, *Haemophilus* and *Capnocytophaga* became depleted in RMGIC group but *Prevotella* enriched. Quantification of *Fusbacterium nucleatum* and *Streptococcus mutans* showed significant difference between RMGIC and RMD groups at T2. Teeth bonded with RMD had significant lower plaque index (PI) and DIAGNODent (Dd) score at T2, compared with teeth bonded with RMGIC (*p* < 0.05). No difference in bracket failure rate was examined between both groups (*p* > 0.05).

**Conclusion:**

By incorporating MPC and DMAHDM into RMGIC, the material could affect the supragingival microbial composition, inhibit the progress of plaque accumulation as well as the key pathogens *S. mutans* and *F. nucleatum* in the early stage of orthodontic treatment.

## Introduction

White spot lesions (WSLs) are the frequently diagnosed side-effect associated with microbial colonization around brackets in fixed orthodontics (Maxfield et al., 2012; Wang et al., 2021). Despite many attempts focusing on the prevention of demineralization, the prevalence of WSLs remains as high as 61% after debonding (Øgaard et al., 2001; Sundararaj et al., 2015). Complex orthodontic apparatus bonded on the teeth obstruct the natural remineralization and oral hygiene maintenance. Irregular surfaces of appliances as well as rough surfaces of adhesives could easily induce biofilm accumulation (Øgaard et al., 1988). Acidogenic and aciduric bacteria in the supragingival plaque thereby actively metabolize sugary diet and induce enamel demineralization (Türköz et al., 2012). Decalcification lesions around brackets have a greater risk forming severe cavities, which could affect aesthetics and patient satisfaction after orthodontic treatment (Maxfield et al., 2012).

WSLs result from increased plaque accumulation due to imbalanced oral hygiene maintenance around orthodontic appliances. During polymicrobial biofilm formation, a key species *Fusobacterium nucleatum* can enhance this process by its powerful ability of adhesion to other bacteria (Palmer et al., 2010). In addition to that, the enrichment of cariogenic bacteria attribute to the enamel demineralization lesions. Evaluation of caries-related bacteria in orthodontic treatment has focused principally on *Streptococcus mutans* (Ahn, Lim & Lee, 2007).

How to effectively prevent these microbial associated side effects has always been a serious challenge faced by orthodontists (Aljohani & Alsaggaf, 2020). Many approaches have been adopted clinically such us mouth rinse, fluoride gel, varnish and sealant (Flynn et al., 2022; Mehta et al., 2015). By using these methods, WSLs and gingivitis have been observed less significant in multiple studies. However, the preventive effect of these methods was reported at low level of evidence in some studies, due to requiring periodical use and heavily relying on patient cooperation (Kirschneck et al., 2016; Sonesson et al., 2021). Therefore, other attempts without treatment compliance were made to improve the effect of WSLs prevention.

Orthodontic adhesive was an option introduced to prevent enamel demineralization without periodic application. Resin-Modified Glass Ionomer Cement (RMGIC) have been reported with better remineralizing effect than resin-composite adhesives due to fluoride-releasing property (Chadwick & Gordon, 1995). However, very limited evidence was confirmed that RMGIC is beneficial in reducing the occurrence of WSLs around brackets compared to resin composite (Khan, Fida & Gul, 2020). The level of F- release from RMGIC could not come up to an effective antimicrobial concentration and that rapidly decrease over time (Lussi & Carvalho, 2015). Therefore, RMGIC is not capable to decrease the WSLs occurrence in terms of combating biofilm formation and inhibiting microbes. It is still quite necessary to further enhance the antimicrobial performance of current orthodontic adhesives.

Salivary protein adsorption and acquired pellicle attachment on the tooth surface is essential for biofilm formation (Chawhuaveang et al., 2021). To combat this process, 2-Methacryloyloxyethyl phosphorylcholine (MPC), an effective methacrylate monomer with a phospholipidpolar group in the side chain, was recently introduced into dental materials (Zhang et al., 2014). MPC containing adhesives could prominently inhibit saliva-derived protein adsorption and biofilm formation on the material surface. Meanwhile, to enhance the antibacterial performance, quaternary ammonium methacrylates (QAMs) were synthesized and introduced into multiple dental materials (Antonucci et al., 2012). Dimethylaminohexadecyl methacrylate (DMAHDM) with an alkyl chain length of 16 was successfully developed and incorporated into dental adhesives, displaying an excellent antibacterial capacity (Zhang et al., 2015b).

Our previous *in-vitro* studies have confirmed that simultaneous addition of MPC and DMAHDM into orthodontic RMGIC could yield significant protein-repellent and antimicrobial effect (Zhang et al., 2015b). (Zhang et al., 2015a) However, no effort has been made to investigate the *in-vivo* performance of any adhesives with these two agents. Therefore, the latest developed novel cement RMGIC + MPC + DMAHDM (referred to as RMD) was firstly applied in this *in-vivo* study. To explore the antimicrobial effect during orthodontic treatment, its influence on microbial community around brackets needs to be studied.

In this study, we aimed to answer three questions: (1) How does RMD affect supragingival microbiome community around brackets during orthodontic treatment? (2) Does RMD reduce the amount of the key pathogen related to caries and biofilm formation? (3) Is RMD more effective to prevent enamel demineralization than conventional RMGIC? To answer these questions, both RMD and conventional RMGIC were studied on the same cohort. Supragingival plaque around adhesives was collected to perform 16S rRNA sequencing and RT-qPCR analysis. Relevant clinical parameters were recorded in the early stage of treatment.

## Materials & Methods

### Study design

This study was approved by the Ethical Committee of Capital Medical University, Beijing, China (IRB No.CMUSH-IRB-KJ-PJ-2022-03). Ten orthodontic patients (six females, four males; age 21.4 ± 3.5 years) were enrolled from Department of Orthodontics, Beijing Stomatological Hospital affiliated to Capital Medical University. Patients were included with full permanent dentition, all teeth with sound enamel, without caries or periodontal disease. Subjects were excluded if they had orthodontic history, required extractions or orthognathic surgery in the treatment plan. Any subject who had systemic diseases, smoking, long-term medication or antibiotics intake within 3 months was also excluded. All recruited subjects were consented, and the written informed consent form were received before treatment.

**Figure 1:**
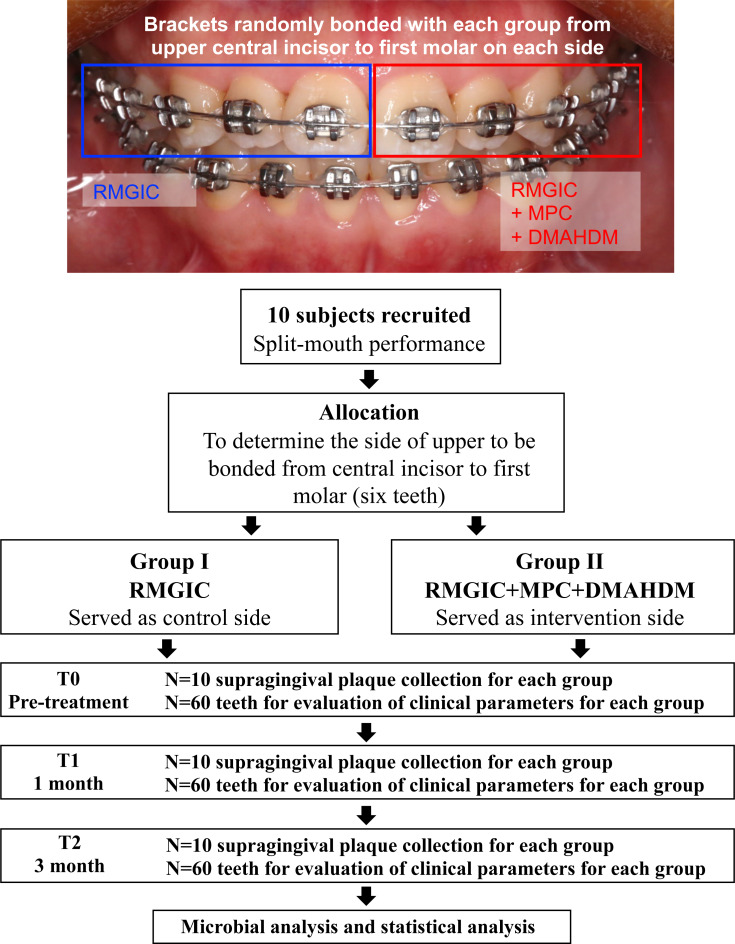
Flowchart of the study protocol.

This study was performed by a split-mouth design shown as Fig. 1. Briefly, for each subject, six teeth in one maxillary quadrant (unilateral central incisor, lateral incisor, canine, premolars and first molar) were bonded with the modified cement RMGIC + MPC + DMAHDM (referred to as RMD), while six namesake teeth on the opposite maxillary quadrant were bonded with commercial RMGIC as control. This self-control design could minimize the variation of oral microbiome profile between individuals. Bracket bonding were consistently carried out by the same operator.

### Cement preparation and brackets bonding

For teeth bonded with RMD, mass fraction of 3% MPC (730114, Sigma-Aldrich, USA) and 3% DMAHDM (prepared according with Makvandi et al., 2018) were carefully weighed then incorporated into RMGIC (Fuji Ortho LC; GC Corporation, Tokyo, Japan) powder phase. Blended powder phase was then mixed with the liquid phase at a mass ratio of 2.5:1. Contralateral teeth for control group were bonded with only RMGIC (Fuji Ortho LC, GC, Japan) mixed with the same powder/liquid ratio.

For all studied teeth, center of clinical crown was firstly cleaned by absorbent cotton roll, then air-dried and acid-etched by 37% phosphoric acid gel (3M Unitek) for 30 s. The etched area was slightly smaller than the base area of the bracket. Either uncured RMD or RMGIC cement was smeared on the bracket base (OPK-A, Tomy, Japan). To make the cement thickness as equal as possible, a 3.0 N force was applied perpendicular to the bonding area for 5 s *via* FGP−0.5 gauge (SHIMPO, Japan). Redundant cement was gently removed by explorer and brackets were then light cured for 40 s. Bonding procedure for all subjects was performed by the same operator. During the period of the study, oral hygiene maintenance of all subjects was normalized by using the same brand of fluoride-free dentifrices. Moreover, subjects were instructed not to use any chewing gum, fluoridated mouthwash or antibiotics, which might have influence on oral microbiome.

### Clinical parameters

To examine any influence of both materials on the development of enamel demineralization or gingivitis, relevant clinical indices were monitored by the same trained clinician at three time points: 1 week before bracket bonding (T0), 1 month (T1) and 3 months (T2) after bracket bonding. At each time point, before oral hygiene and plaque collection was performed, Turesky modification of the Quigley and Hein plaque index (Turesky QH PI) was used to assess the level of plaque accumulation around brackets (Löe, 1967).

To evaluate demineralization around brackets, enamel decalcification index (EDI) was examined by visualization. EDI score was calculated according to a previous study (Banks & Richmond, 1994). Briefly, for each subject, each quadrant area around the bracket (mesial, distal, occlusal and gingival) from the six teeth was scored for decalcification level. EDI of each subject was averaged equal to the total scores divided by the total number of quadrants. In addition, decalcification severity of the teeth was quantified with the laser fluorescence device DIAGNOdent pen 2190 (Kavo, Biberach, Germany) and scored the same way as EDI (Mizrahi, 1982; Aljehani, Yang & Shi, 2007b). The cylinder sapphire tip designed for use on occlusal surfaces was placed onto the device when performing the measurements. DIAGNOdent readings (Dd) was recorded at each quadrant zone one mm off the bracket with three replicates. Dd score of each tooth was the average of four quadrants. The score of each individual was the average of all bonded teeth. Meanwhile, the measurements of gingival index (GI) related to gingivitis development were performed with the use of a periodontal probe according to standard procedures (Katz et al., 2002; Loe, Theilade & Jensen, 1965).

Besides, bracket bonding failures were also monitored at each visit. All studied teeth were checked for bracket debonding, and the number of bracket failures was recorded at each visit. De-bonded brackets were thoroughly sprayed to remove the residual adhesive and replaced with the same type of adhesive. Bracket failures had been followed up for three months. Data on bonding failures were collected for each subject to compare the *in-vivo* bonding strength and stability of both types of cement.

### Supragingival plaque collection

Supragingival plaque samples were collected at all three timepoints described above right after the recording of clinical parameters. Subjects were required not to perform any oral hygiene for at least 12 h before sampling. Saliva was firstly removed by gently gargled with warm water then air-drying, that not disturbing the sampling area. Supragingival plaques around brackets were scraped off by the same trained clinician using a sterile Gracey curette 7/8. Plaques from six teeth bonded with the same material were pooled together as one sample in a 1.5 ml microcentrifuge tube containing 300 ul 1× PBS on ice. Therefore, two tubes of plaque samples, namely one tube of RMGIC and one tube of RMD, were obtained from the same subject at each time point. Samples were then centrifuged at 12,000 × g for 5 min. Supernatant was gently moved out by pipette and the pellet was stored at −80 °C for further use.

### DNA Extraction

Total bacterial genomic DNA from the supragingival plaque was extracted and purified using QIAamp DNA Mini Kit (Qiagen Sciences, Valencia, CA, USA) following the manufacturer’s instructions. The DNA quality and quantity were measured using a Nanodrop 2000 spectrophotometer (Thermo Scientific, Waltham, MA, USA) and checked on 1% agarose gels. All samples with DNA concentration higher than 50 ng/ul, and the optical density of A260/A280 ratios between 1.8−2.1 were stored in 1 × Tris-EDTA (pH = 8.0) at −80 °C. Eluted DNA was further used for the amplicon sequencing and qPCR analysis.

### 16S rRNA amplicon sequencing and bioinformatic analysis

PCR amplification of the 16S ribosomal RNA gene V3-V4 region was performed using the specific primers 341F (5′-CCTACGGGNGGCWGCAG-3′; 806R (5′-GGACTACHV GGGTATCTAAT-3′) with a unique eight-base barcode to each sample (Jiang et al., 2016). PCR products were separated by 2% agarose gel electrophoresis and ligated to construct a sequencing library according to the manufacturer’s instructions (NEXTFLEX Rapid DNA-Seq Kit; PerkinElmer, Waltham, MA, USA). Purified amplicons were pooled in equimolar and sequenced with 2 × 250 paired-ends on an Illumina Miseq platform (Gene Denovo, Guangzhou, China). The raw sequences obtained have been submitted to the NCBI Sequence Read Archive (SRA) database under accession number SRP405005.

After demultiplexing and trimming the barcodes, raw sequences with low-quality or uncertain base pairs were filtered and removed by QIIME (v 1.9.1). Clean sequences were then clustered into operational taxonomic units (OTUs) at a 97% similarity cutoff using USEARCH (v 9.2.64). The taxonomy of each 16S rRNA gene sequence was assigned to the Human Oral Microbial Database (HOMD) (Dewhirst et al., 2010). Shannon_e and Simpson indices were used to evaluate the alpha-diversity, and PCoA based on weighted_unifrac distance was conducted to assess the beta-diversity (Gazdeck et al., 2019). Relative abundance was assessed to compare the microbial composition between groups.

### RT-qPCR

To validate the absolute abundance of two critical pathogens in the process of biofilm formation and caries development, the quantification of *S. mutans*, and *F. nucleatum* in the plaque was evaluated using RT-qPCR as previously described (Childers et al., 2011; Tanner et al., 2012). Briefly, *S. mutans* (UA 159) cultivated in Brain Heart Infusion (BHI) and *F. nucleatum* (ATCC 25586) cultivated in Columbia Broth (CB) anaerobically at 37 °C were used to establish a standard curve, respectively. Cultures in late logarithmic growth (ODAb600 = 1.0) was 10-fold diluted on BHI (*S. mutans*) and CB (*F. nucleatum*) agar plates. Viable counts (CFU/ml) numerated from the plates were well associated with 10-fold serial dilutions of extracted DNA from each species by linear regression curve for standardization. Primers for *S. mutans* (F: 5′-GCCTACAGCTCAGAGATGCTATTCT-3′, R: 5′-GCCATACACCACTCATGAATTGA-3′) and *F. nucleatum* (F: 5′-GGCCACAAGGGGACTGAGACA-3′, R: 5′-TTTAGCCG TCACTTCTTCTGTTGG-3′) were used in the reaction mix system of 20 µL. The reaction mix comprised of 0.5 µM of each primer with 1X SYBR Green Master Mix (Bio-Rad, Hercules, CA, USA) and 20 ng of DNA. A standard dilution series of known amounts of genomic DNA from 2 ng/µL to 20 fg/µL were assessed in the same assay. Cq-values obtained from the standards were used to generate a standard curve from which the amount of DNA in the unknown samples. A regression analysis was performed and thus quantify the corresponding concentrations of genomic DNA of each target. Real time PCR was performed as the following condition: initial denaturation for 3 min at 94 °C; 40 cycles of denaturation for 5 s at 94 °C, annealing for 15 s at 59 °C/58 °C; extension for 10 s at 72 °C. The DNA concentrations of all unknown samples were obtained then transformed to CFU/mL by regression analysis using the standard curve.

### Statistical analysis

For data with homogeneity of variance, Student *t*-test or one-way ANOVA was performed, otherwise, a Wilcoxon rank-sum test was applied using SPSS 25.0. Comparisons of alpha and beta diversity were performed using the Kruskal–Wallis H test. Differences in relative abundance between multiple groups were analyzed using the Wilcoxon rank-sum test. A statistically significance criterion was defined as *p* < 0.05.

## Results

We monitored the bracket failure bonded with both RMGIC and RMD materials in the first six months. The failure rates of both materials at the two checkpoints were all below 5% (Table 1). There was no significant difference between RMGIC and RMD at each time point (*p* > 0.05), which indicated RMGIC incorporating both 3% MPC and 3% DMAHDM had excellent clinical bonding performance.

**Table 1:** Bracket failures of both materials at indicated time points (*n* = 60).

Time point	Number (rate) of bracket failures		*χ* ^2^	*p* value
	RMGIC (*n* = 60)	RMD (*n* = 60)		
T1	2 (3.3%)	3 (5%)	0.209	0.647
T2	1 (1.7%)	1 (1.7%)	0	>0.999

**Figure 2:**
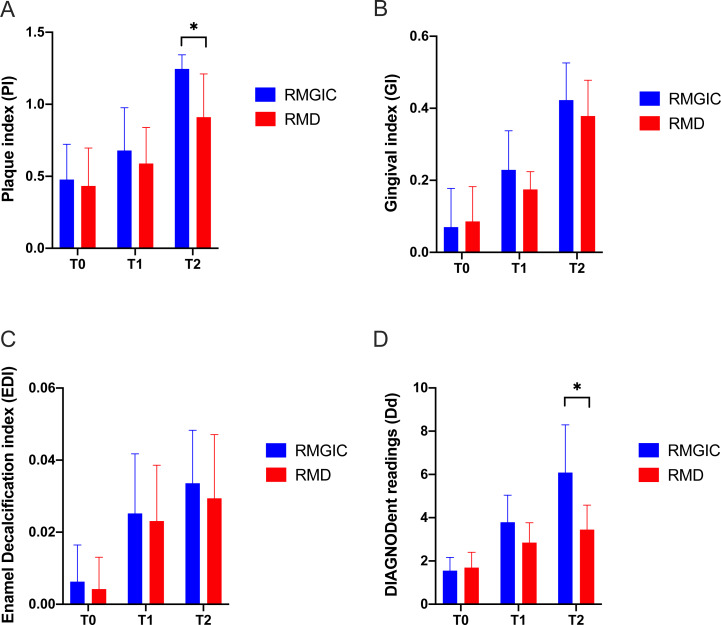
Comparison of clinical parameters bonded with RMGIC and RMD at all time points. (A) Plaque index (PI), (B) gingival index (GI), (C) enamel decalcification index (EDI), (D) DIAGNODent readings (Dd). Bar plots were presented as the mean with standard error. * *p* < 0.05.

In the first three months, relevant clinical parameters were recorded on schedule. As shown in Figs. 2A–2D, all indices had an increasing trend from T0 to T2. Much slower increasing in plaque accumulation was observed in RMD rather than RMGIC (Fig. 2A). From T0 to T2, EDI on both groups had a slight upward but insignificant difference at each time point (Fig. 2C). Dd score on RMGIC group had a more pronounced increase than RMD. Although the average Dd scores on both groups remained below the threshold for diagnosable demineralization (Aljehani, Yang & Shi, 2007a) during the observation period, RMGIC still had a prominent higher reading than RMD at T2 (*p* < 0.05, Fig. 2D). Same trend could be seen in GI, while the GI scores on both groups were also below the threshold for diagnosing gingivitis after three months (Fig. 2B).

In terms of the *in-vivo* effect of both materials on oral bacteria, we firstly compared the supragingival microbiome change around brackets *via* 16S rRNA sequencing. A total of 395,143 reads were obtained from 60 plaque samples. After filtering poor-quality reads and mapping with HOMD database, an average of 55,403 clean reads and 258 OTUs per sample was obtained. Among all detected OTUs, 208 uniform OTUs were identified from both groups.

**Figure 3:**
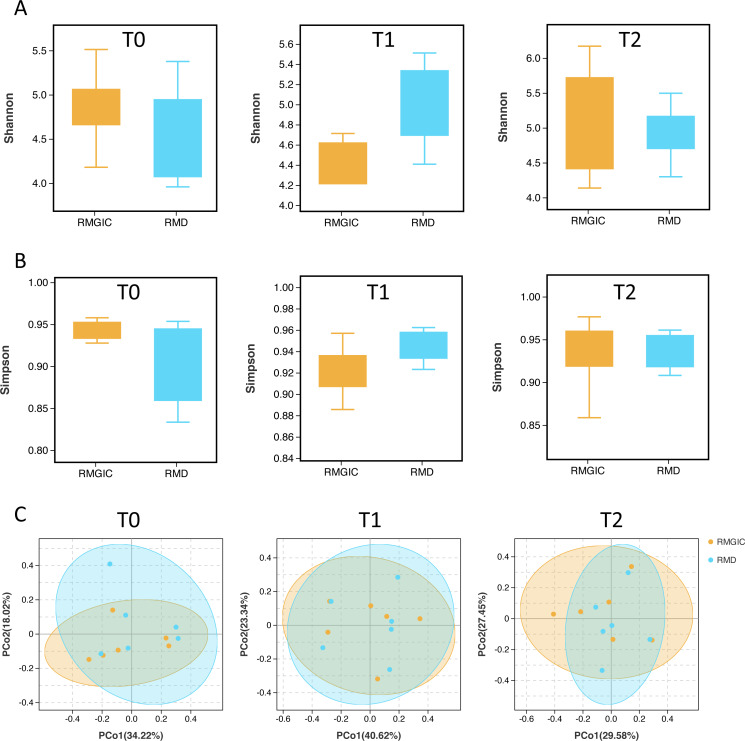
Alpha and beta diversity analysis of RMGIC and RMD group at all time points. (A) Shannon_e index; (B) Simpson index; (C) beta diversity calculated *via* weighted unifrac distance.

We compared the Shannon and Simpson indices of alpha diversity between both groups from T0 to T2. The alpha diversity didn’t significantly change over time either in RMGIC or RMD group (Fig. 3A). There was no difference for all the alpha diversity indices between both materials, which indicated that the intra-group microbial diversity basically remained stable in the first three months. Meanwhile, at each timepoint, comparison of the beta diversity *via* weighted unifrac distance suggested the sample population of RMD group was close to RMGIC group at each time point (Fig. 3C).

**Figure 4:**
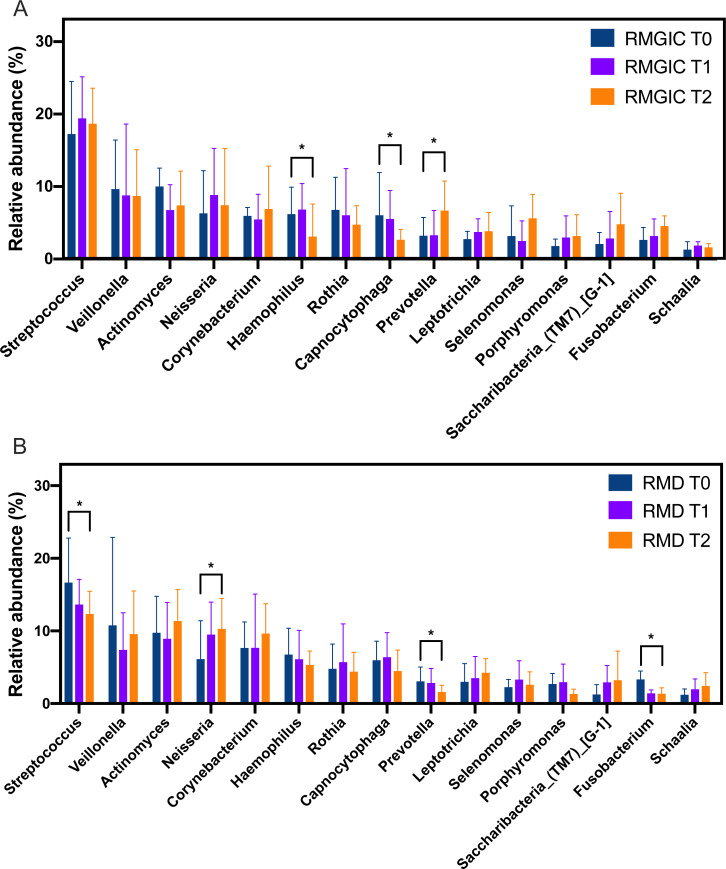
Relative abundance at genus level from supragingival plaque in RMGIC and RMD groups. (A) RMGIC group, (B) RMD group. Top fifteen abundant taxa were plotted at indicated time points. Asterisk indicated statistical significance was examined by Wilcoxon rank-sum test. * *p* < 0.05.

Following the analysis of microbial diversity, we next compared the microbial profile in supragingival community. Although the overall microbial composition was similar between both groups, a few of core members within oral microbiome still exhibited a slight but differentiated shifting trend. Abundance of the top 15 abundant genera were listed in Fig. 4, and comparison of each taxa among different timepoints was performed. From T0 to T2, several gram-negative bacteria such as *Fusobacterium*, *Prevotella*, *Selenomonas* and *Porphyromonas* showed a trend of enrichment in RMGIC group but depleted in RMD group. Interestingly, this trend was not significant in most of gram-positive bacteria except for *Streptococcus*. In the RMD group, *Streptococcus*, *Prevotella* and *Fusobacteirum* accounted for fewer abundance at T2 (*p* < 0.05) than T0, while the relative abundance of *Nesseria* became higher. However, in the RMGIC group, a higher abundance of *Prevotella* was observed at T2 compared to T0 (*p* < 0.05).

**Figure 5:**
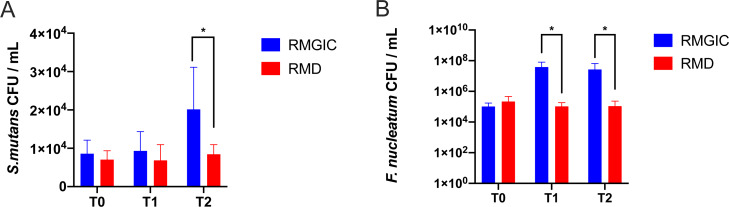
Quantification of *S. mutans* and *F. nucleatum* in supragingival plaque. (A) Result of *S. mutans* at all time points, (B) result of *F. nucleatum* at all time points. *p < 0.05.

Although we observed the decreasing trend in some gram-negative bacteria in RMD material, the amount of key pathogen for biofilm formation and dental caries still required further comparison between both groups at species level. *S. mutans* and *F. nucleatum* were detected by qPCR in all thirty-six samples. There was no statistical difference in *S. mutans* CFU at T0 (*p* > 0.05), indicating the initial load of *S. mutans* in the plaque was basically consistent on both sides before treatment. From T0 to T2, the absolute abundance of *S. mutans* in RMGIC group remained steady but slightly depleted in RMD group (Fig. 5A). The amount of *S. mutans* was significantly higher in RMGIC than RMD at T2 (*p* < 0.05).

Interestingly, unlike *S. mutans*, the *F. nucleatum* CFU in the plaque had a different changing pattern between RMGIC and RMD materials (Fig. 5B). Despite both groups had a parallel baseline level at T0, *F. mucleatum* around RMGIC cement prominently increased at following time points, while only mildly increased around RMD cement. The *F. nucleatum* CFU in RMGIC group was remarkably higher than that of RMD group at T1 and T2 (*p* < 0.05). These results indicated the addition of MPC and DMAHDM into RMGIC cement could induce the change of microbial composition in the first three months.

## Discussion

Enamel demineralization as well as gingivitis are common microbial induced side-effects affecting dental hard and soft tissue in fixed orthodontics (Tanner et al., 2012). Placement of orthodontic appliances increased the difficulty in oral hygiene maintenance. Biofilm accumulation around brackets could easily accelerated the development of diseases (Atassi & Awartani, 2010). Fluoride-containing RMGIC is currently widely used but still not enough to inhibit biofilm formation as well as enamel demineralization (Rogers, Chadwick & Treasure, 2010). Therefore, efforts have been made to develop novel antimicrobial materials for brackets bonding. Zhang et al. (2014)firstly reported the protein adsorption on material surface was significantly restrained by incorporating MPC into dental adhesives. Novel quaternary ammonium methacrylate DMAHDM was confirmed with strongest antibacterial property in its QAMs family (Zhou et al., 2013). Numbers of following studies showed that the *in-vitro* performance on biofilm and bacterial inhibition by simultaneously incorporating MPC and DMAHDM into multiple dental materials was prominent (Zhang et al., 2015a; Zhang et al., 2015c; Zhang et al., 2018). MPC and DMAHDM could synergistically combat microbes by inhibiting the attachment of salivary protein and bacterial aggregation (Zhang et al., 2018). However, to date the *in-vivo* effect of these two promising agents is still undefined due to lack of *in-vivo* study.

This pilot study firstly investigated the clinical effect of RMGIC incorporated with both MPC and DMAHDM in the early stage of fixed orthodontics. To better compare the influence of modified adhesive to unmodified material, we monitored the change of supragingival microbiome around the brackets bonded with either modified cement (RMD) or unmodified cement (RMGIC) *via* split-mouth performance to the same cohort. This design has been adopted in multiple studies relevant to oral hygiene or microbial change, which could lessen the individual variation of oral microbiome as much as possible (Pellegrini et al., 2009; Pretti et al., 2015).

In the results of this study, adding MPC and DMAHDM together did not compromised the clinical bonding performance within the observation period. Previous studies suggested the coefficient proportion of mixing MPC and DMAHDM together into dental materials could be 3% respectively (Wang et al., 2016). Under this adding proportion, the mechanical property of RMGIC was not compromised. Our preliminary study also confirmed the addition RMGIC with 3% MPC and 3% DMAHDM together did not affect bonding strengths after 6-months water-aging (data not shown). Therefore, RMD cement in this study was proved reliable for long-term application in real oral environment during orthodontic treatment.

Regarding the antimicrobial effect, our results indicated that both materials had different impact on supragingival microbiome. The alpha and beta diversity in the plaque did not significantly change either in the RMD or RMGIC group, which demonstrated that the MPC and DMAHDM had no significant effect on the diversity structure of oral microbes. The variety of oral microbiota in the supragingival plaque basically remained stable in the first three months of orthodontic treatment, which was also consistent with the previous report (Campobasso et al., 2021). Although the overall oral microbial community was not significantly altered, the relative abundance results showed that different members in the community diffrently on both groups. RMD had a significant inhibition on the increasing trend of several gram-negative bacteria such as *Prevotella* spp., *Porphyromonas* spp., and *Fusobacterium* spp. These bacteria were commonly detected enriched in periodontal disease. In terms of the effect of RMD on gram-positive bacteria, no obvious inhibition was observed on some major genera such as *Actinomyces* spp., *Corynebacterium* spp., *Rothia* spp. and *Corynebacterium* spp. except for *Streptococcus* spp. In RMGIC group, an increasing trend on gram-negative bacteria like *Prevotella* spp. and *Fusobacterium* spp. could be detected, but not observed on *Haemophilus* spp. and *Capnocytophaga* spp. in the RMGIC group. A recent study reported that in the early stage of orthodontic treatment, gram-negative anaerobes could become enriched in supragingival plaque while gram-positive bacteria might depleted (Campobasso et al., 2021). In our study, similar shifting trend was observed on many genera in the RMGIC group but not in the RMD group. Inhibition on gram-negative bacteria was more noticeable in RMD compared to RMGIC. These results indicated that RMD might have more influence on gram-negative bacteria than gram-positive bacteria. In terms of the species we studied, *F. nucleatum* prominently increased in the RMGIC group but inconspicuous in the RMD group, which was consistent with the change in genus level. Interestingly, an inhibition on *S. mutans* increasing was also observed in the RMD group compared to the RMGIC group.

The reason for this outcome was possibly associated with the antimicrobial mechanism of DMAHDM. Quaternary ammonium compounds can penetrate the bacterial cell wall and membrane, causing the leakage of cytoplasmic content (Zhou et al., 2013). These compounds also have positively charged N+ quaternary amine in the structure of that contacting the negatively charged bacterial membrane, causing the loss in the balance of essential ions as well as disturbance in the membrane functions (Melo et al., 2013; Zhou et al., 2013). Long-chain compounds DMAHDM might be more difficult to penetrate the gram-positive bacteria, which have much thicker peptidoglycan cell wall (Zhou et al., 2013). This might contribute to the difference influence of both materials on different taxa of bacteria. However, this speculation needs larger sample size to be investigated in future.

In this study, we also monitored clinical parameters related to plaque accumulation, demineralization and gingivitis. The Dd score in the RMD group showed that the level of demineralization was lower than that in the RMGIC group, although the results of EDI and Dd scores was not sufficient to diagnose demineralization on both groups after 3 months. Previous studies have found that various periodontal indices increased after 3 months of treatment, among which the increase in plaque index was the most obvious (Kozak, Lasota & Chałas, 2021). In our study, compared to RMGIC, plaque accumulation was significantly inhibited by RMD, while no significant difference was observed on gingival index. This indicates that RMD successfully inhibited biofilm formation during the early stage of treatment. These results can be initially explained by the hydrophilic property of MPC that modifies the hydrophobic surfaces on material to reduce proteins adsorption (Katsikogianni & Missirlis, 2004). Thus, by repelling the salivary proteins layer MPC might contributes to increasing the interaction between DMAHDM and bacteria, which reinforces the antibacterial effect (Zhang et al., 2015a).

The gingival health on both groups was not distinguishable in the initial stage of treatment. RMD was still expected beneficial to maintain better periodontal health due to its effective inhibition on gram-negative pathogens. Previous *in-vitro* study also confirmed the ability of MPC and DMAHDM incorporated materials on the constraint of periodontal pathogens (Wang et al., 2016). It is especially worthwhile when the orthodontic appliance is bonded close to gingival margin. However, to validate the antimicrobial and clinical preventive effect on orthodontic associated oral disease, this study still needs to get more subjects enrolled and monitor with longer period.

To answer the questions we proposed in this study, firstly, RMD could yield different changes on supragingival microbiome around brackets but no significantly altering microbial diversity. RMD was effective to inhibit the increase of *S. mutans* and *F. nucleatum* in the early stage of the treatment. Compared to conventional RMGIC, RMD could yield excellent performance combating plaque accumulation. It was difficult to draw any conclusion whether RMD was more effective to prevent WSLs within a 3-months observation period. The results of this study suggested that the novel MPC and DMAHDM modified orthodontic cement was promising in WSLs prevention by affecting biofilm formation and microbial community. However, regarding to the limited sample size of this study, a convincing conclusion cannot be drawn. Moreover, a split-mouth design could minimize the inter-individual difference, but it might introduce intraoral interaction of microbiome between both groups. A parallel randomized trial with larger sample size and longer observation is required in the future study.

## Conclusions

In conclusion, the addition of both 3% MPC and 3% DMAHDM into RMGIC did not compromise clinical bonding property. In the early stage of orthodontic treatment, the modified RMD material could effectively reduce the accumulation of supragingival plaque around brackets. Microbial composition of supragingival microbiome was affected to a certain extent. Its preventive effects on enamel demineralization still need to be further investigated.

##  Supplemental Information

10.7717/peerj.14820/supp-1 Supplemental Information 1 Raw data: Fig. 2 Clinical parameters

10.7717/peerj.14820/supp-2 Supplemental Information 2 Raw data of CFU results

## Additional Information and Declarations

### DNA Deposition

The following information was supplied regarding the deposition of DNA sequences: The 16S rRNA sequences are available at NCBI: PRJNA890880.

### Data Availability

The following information was supplied regarding data availability: Raw data are available in the Supplemental Files. The sequence data is available at NCBI Sequence Read Archive (SRA): SRP405005.
